# Editorial: Individual neurodynamics tunes personalized neuromodulation [INTuNe]

**DOI:** 10.3389/fnins.2024.1508578

**Published:** 2024-11-26

**Authors:** Franca Tecchio, Karolina Armonaite, Elzbieta Olejarczyk

**Affiliations:** ^1^Laboratory of Electrophysiology for Translational Neuroscience, Institute of Cognitive Sciences and Technologies, National Research Council, Rome, Italy; ^2^Faculty of Engineering, Uninettuno University, Rome, Italy; ^3^Nalecz Institute of Biocybernetics and Biomedical Engineering, Polish Academy of Sciences, Warsaw, Poland

**Keywords:** neurodynamics, neuromodulation, functional source separation (FSS), transcranial alternating current stimulation (tACS), closed-loop

In contemporary neuroscience, the nervous system is increasingly recognized as a complex, dynamic entity, driven by the interplay with the environment of various interacting sub- components. These elements operate across multiple spatiotemporal scales, supporting continuous feedback between individuals and their environments. Within this framework, neural networks consist of interconnected nodes that process, relay, and interpret information depending on the pursued goal. The activity within these networks, particularly during the resting state, serves as a critical indicator of neural health and overall brain function.

Neurodynamics, the temporal patterns of electrical activity of neuronal pools, has become essential for understanding how intrinsic brain activity translates into behavioral and cognitive functions. During rest, these patterns reflect the neural network's intrinsic properties, emerging from the complex interplay of different brain regions as result of development and learning. Recent technological advances have enabled unprecedented precision in capturing these dynamics, making it possible to observe and manipulate even subtle fluctuations in electrical activity (Gorgey et al., Carè et al.). This capacity has profound implications for understanding and treating various neurological and psychiatric conditions, from epilepsy to post-traumatic stress disorder, behavioral disorders, depression, fatigue and beyond. The four contributions developing the “*Individual Neurodynamics Tunes Personalized Neuromodulation*” Research Topic highlight how the synergy between recording neuronal pool activity, stimulating neural tissue, and supporting sensory perception and motor control through neuroprosthetics can revolutionize the lives of individuals with severe impairments. Furthermore, understanding the “language” spoken by the neuronal pools in our brain allows us to innovate the interaction of our body with the environment, in the individual, social and physical-architectural dimensions.

Technological innovations in microelectronics, ultrasound, and optical sensors have provided new ways to record, amplify, and interpret brain activity. These advances have significantly broadened our ability to interact with the nervous system in a targeted manner. Importantly, the use of techniques such as magneto- and electro- encephalography (MEG and EEG) and Functional Source Separation (FSS) allows researchers to investigate the spatial and temporal complexity of neural dynamics with great accuracy (Pascarella et al.).

For example, studies using Normalized Compression Distance (NCD) have provided an assessment of functional homology between brain hemispheres in the resting state, offering insights into how neurodynamics can reflect structural and functional connectivity (Pascarella et al.). This work underscores the importance of understanding individual variability in neurodynamics and how these patterns change with age or neurological conditions, such as depression and fatigue. By recognizing these patterns, and deepening the understanding of the relationship between hemispheric homologs, we can better tailor treatments to individual patients, offering a path toward more effective and personalized therapeutic interventions.

The field of neuromodulation is one of the most promising areas in the treatment of neurological disorders. While non-invasive brain stimulation (NIBS) techniques have shown great potential, their effectiveness can vary significantly between individuals. This variability underscores the need for personalization in neuromodulation strategies, mirroring advances in personalized pharmacotherapy. By leveraging individual neurodynamics, clinicians can develop neuromodulation protocols that are finely tuned to the specific needs and characteristics of each patient (Carè et al.). Such personalized approaches are particularly promising in chronic conditions like addiction, epilepsy, and depression, where traditional treatments have often fallen short.

Closed-loop neuroengineering systems, which monitor and adapt to real-time changes in brain activity, represent the next frontier in personalized neuromodulation. These systems have the potential to dynamically adjust stimulation parameters based on the individual's ongoing neurodynamics, thereby improving therapeutic outcomes (Carè et al.). For instance, recent studies have shown that transcranial alternating current stimulation (tACS) can modulate functional connectivity between brain regions in a manner dependent on the intensity and frequency of stimulation (Wansbrough et al.). Such findings highlight the importance of aligning stimulation parameters with the individual's intrinsic brain neurodynamics to optimize the therapeutic effect.

The future of personalized neuromodulation lies in our ability to harness the rich, dynamic information embedded within individual neurodynamics. By combining cutting-edge technology with a deeper understanding of how neural networks function at both local and global scales, we can develop highly specific and effective treatments for neurological and psychiatric disorders. This approach holds the promise of not only alleviating symptoms but also promoting long-term recovery and enhancing quality of life for patients.

As research continues to evolve, the integration of neurodynamic information into neuromodulation protocols will undoubtedly play a critical role in shaping the next generation of personalized brain therapies.

